# Effect of Advanced Glycation End-Products and Excessive Calorie Intake on Diet-Induced Chronic Low-Grade Inflammation Biomarkers in Murine Models

**DOI:** 10.3390/nu13093091

**Published:** 2021-09-02

**Authors:** Matheus Thomaz Nogueira Silva Lima, Michael Howsam, Pauline M. Anton, Carine Delayre-Orthez, Frédéric J. Tessier

**Affiliations:** 1 University Lille, Inserm, CHU Lille, Institut Pasteur de Lille, U1167—RID—AGE—Facteurs de Risque et Déterminants Moléculaires des Maladies Liées au Vieillissement, F-59000 Lille, France; matheusthomaz.nogueirasilvalima@univ-lille.fr (M.T.N.S.L.); michael.howsam@univ-lille.fr (M.H.); 2 Institut Polytechnique UniLaSalle, Université d’Artois, ULR 7519, Equipe PETALES, F-60026 Beauvais, France; pauline.anton@unilasalle.fr (P.M.A.); carine.delayre@unilasalle.fr (C.D.-O.)

**Keywords:** chronic low-grade inflammation, diet, advanced glycation end-products, metabolic diseases, high-fat diet, carboxymethyllysine

## Abstract

Chronic Low-Grade Inflammation (CLGI) is a non-overt inflammatory state characterized by a continuous activation of inflammation mediators associated with metabolic diseases. It has been linked to the overconsumption of Advanced Glycation End-Products (AGEs), and/or macronutrients which lead to an increase in local and systemic pro-inflammatory biomarkers in humans and animal models. This review provides a summary of research into biomarkers of diet-induced CLGI in murine models, with a focus on AGEs and obesogenic diets, and presents the physiological effects described in the literature. Diet-induced CLGI is associated with metabolic endotoxemia, and/or gut microbiota remodeling in rodents. The mechanisms identified so far are centered on pro-inflammatory axes such as the interaction between AGEs and their main receptor AGEs (RAGE) or increased levels of lipopolysaccharide. The use of murine models has helped to elucidate the local and systemic expression of CLGI mediators. These models have enabled significant advances in identification of diet-induced CLGI biomarkers and resultant physiological effects. Some limitations on the translational (murine → humans) use of biomarkers may arise, but murine models have greatly facilitated the testing of specific dietary components. However, there remains a lack of information at the whole-organism level of organization, as well as a lack of consensus on the best biomarker for use in CLGI studies and recommendations as to future research conclude this review.

## 1. Introduction

Diet plays a role in the induction and progression of Chronic Low-Grade Inflammation (CLGI) which has been associated with metabolic diseases such as obesity and diabetes [1]. Some food contaminants such as the exogenous Advanced Glycation End-Products (AGEs), produced in thermally processed products, have been shown to contribute to the persistent inflammatory component of diabetes, aging, and heart failure [2]. The same AGEs can also be formed at 37 °C, being called endogenous AGES. The deleterious effects of higher circulating levels of AGEs on health may be mediated by their eponymous cell membrane receptor RAGE [3]. Nonetheless, the overconsumption of macronutrients also associated with “western”, processed foods, such as lipids and carbohydrates, plays a similar role in triggering the CLGI implicated in obesity and neuroinflammation, both at the local and systemic levels [4]. The consequences of high lipid intake, for instance, range from appetite dysregulation in the hypothalamic core to metabolic endotoxemia [5], with this last being mediated by toll-like receptor 4 (TLR4) [6]. 

The clinical differentiation of CLGI is still a matter of significant debate. The main biomarkers currently used in the diagnosis of inflammation, such as C-reactive protein (CRP), are not specific to CLGI. Hence, many studies in recent decades have attempted to define CLGI biomarkers in humans and rodent models, including the proposition that clusters of biomarkers may best define the state [7]. While Calder and colleagues [7,8,9] have published comprehensive reviews on CLGI in nutritional studies in humans, detailing the biomarkers used to assess inflammation, important discoveries have concomitantly been made in murine models: to our knowledge, however, no review has yet been published on this body of work. Our goal here is to summarize current discoveries on diet-induced CLGI and discuss the factors which influence CLGI biomarker expression in rodent models.

Although centered on diet-induced CLGI in murine models, we begin our review by considering the essential concepts of acute and chronic inflammation in order to clearly define CLGI. Thereafter, those CLGI biomarkers currently explored in the literature are presented so as to demonstrate their use in such studies, their diversity, and their limitations, and to frame the current understanding of diet-induced CLGI. The corresponding literature on diet-induced CLGI is then reviewed, including dietary AGE consumption and obesogenic diets, to elucidate the current directions in CLGI research and explore the multiple, concurrent events which shape CLGI in rodent models. Firstly, we will explore the effects of immoderate consumption of AGEs on CLGI promotion. In the following sections, the effects of high-calorie diets on CLGI and their implications for adiposity, and the neuroinflammation associated with appetite control will be presented. Findings on gut microbiota remodeling, metabolic endotoxemia, and CLGI initiation will also be discussed. Finally, perspectives on CLGI research gaps that remain to be filled will be addressed.

## 2. Inflammation and Chronic Low-Grade Inflammation (CLGI)

Inflammation is part of the body’s innate and adaptive immune defenses. It comprises a series of cellular and chemical signal barriers which aim to control and conquer endogenous and exogenous stimuli (essentially bacterial or viral infections) and trauma-related damage [10]. Inflammation can be either an acute or chronic process, but both have common aims: namely, to neutralize the source of injury, promote tissue repair, and drive a self-limited return to homeostatic conditions [11]. In contrast to acute inflammation (AI), chronic inflammation (CI) is a process linked to resolution failure and induces continuous recruitment of the cellular immune apparatus, promoting tissue damage [12].

Beyond the difference in duration between AI and CI, the degree of inflammatory response also determines whether inflammation becomes pathological. Under a non-overt inflammation scenario, a chronic, but low-grade inflammatory mechanism comprehends excessive metabolic stress correlated to the rise of circulating levels of inflammation signals [13]. Both CI and CLGI are borderline conditions sharing similar molecular mechanisms, but a distinct involvement of metabolic tissues characterizes CLGI progression. Indeed, CLGI is currently considered to be a possible factor in the pathological aggravation of obesity, type 2 diabetes mellitus (T2DM), atherosclerosis, or cancer [1,14]. 

Compared to the chronic but severe inflammation present in arthritis or Crohn’s disease [15], metabolic disorders (such as obesity) and some age-related conditions (such as frailty) have only a CLGI component [16]. This difference in the intensity of inflammation has orientated research seeking to characterize specific molecular patterns and biomarker clusters in order to develop predictive tools aimed at reducing the health and socioeconomic impacts of these pathologies. 

The discovery and elucidation of the CLGI process was an important step in the comprehension of diseases currently considered to be global public health concerns (e.g., T2DM). The term “low-grade inflammation” was first used in the 1980s with reference to histologic samples from schizophrenic patients in which increased glial fibrils were observed [17], and to the onset of ocular complications in arthritis patients [18]. However, a more complete body of evidence later emerged with the inclusion of tissues previously considered inactive, which today we understand to play a fundamental role as active metabolic organs (e.g., adipose tissue, skeletal muscles, brain) [19]. Hotamisligil and colleagues [20] were the first to report increased expression of TNF-α (both mRNA and protein) in the white adipose tissue of obese mice (ranging from 34 to 166 pg/mL), demonstrating that it participated in the aggravation of obesity and diabetes by increasing insulin resistance [20]. Similarly, the expression of adipokines by visceral fat was later confirmed in humans [21,22].

**Figure 1:**
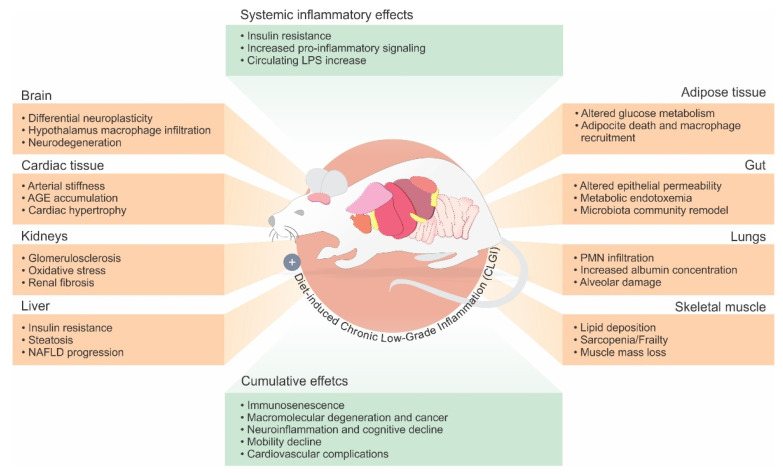
Physiological alterations resulting from diet-induced CLGI in different organs. CLGI is a complex crosstalk among different organs leading to an increase in local (tissue-specific) and circulating levels (systemic inflammatory effects) of inflammatory mediators. Organs and respective inflammatory mediators are referenced in Table 1. As a result, lifelong exposure to CLGI is also related to cumulative effects such as, macromolecular degeneration, neuroinflammation, and mobility reduction associate with age-related diseases with LGI components (Alzheimer’s disease). Such physiological modifications are related to the activation of inflammation, and tissue dysfunction. NAFLD = nonalcoholic fatty liver disease; PMN = polymorphonuclear leukocytes.

Screening for systemic effects of inflammation has demonstrated that several other organs are implicated in CLGI (Figure 1). In the brain, for instance, the hypothalamus plays a role in the control of appetite, regulating the intake of macronutrients and energy [23]. Both hypothalamic macrophage infiltration and TNF-α increase were observed in obese mice [24], and increased gliosis in humans was evidenced from retrospective studies of magnetic resonance records of obese patients that had undergone pituitary or epilepsy imaging protocols [25]. In addition, other key molecular events act as a trigger for CLGI. The accumulation of molecular degenerations, mitochondrial dysfunction, DNA damage, and immune dysregulation are all age-related factors implicated in CLGI [26]. T-cell immunosenescence, for instance, has been demonstrated in visceral fat from mice: adiposity led to an increased T-cell population with senescent molecular profiles expressing programmed cell death protein 1 (PD-1) and tumor necrosis factor ligand superfamily member 8 (TNFSF8) [27,28]. 

More recently, some evidence appears to suggest that the gut microbiota also plays a critical role in CLGI progression, following remodeling as a result of nutritional imbalances. The consumption of increased quantities of fats and/or carbohydrates has been demonstrated to modify the composition of the microbiota and promote permeability of the gut–mucosal barrier [29,30]. A result of this process is increased circulating levels of endotoxin leading to systemic endotoxemia [29]. Taken together, these data suggest that diet-induced CLGI results from the convergence of several conditions, gut dysbiosis and endotoxemia, adipocyte hypertrophy and death, skeletal muscle oxygen use, and hypothalamic macrophage infiltration being key among them (Figure 1). However, the underlying mechanisms of such a complex interaction remain to be fully elucidated.

Although the terms “Low-Grade Inflammation” or “Chronic Low-Grade Inflammation” are used interchangeably in the literature, we here take “chronic” to be a compulsory requirement for the low-grade inflammatory stimulus to promote some sort of pathological effect. Thus, based on the extensive literature currently published, CLGI can be formally defined as a pathological state lacking overt inflammation, but characterized by continuous and unresolved activation of inflammation mediators. It results in increased production of cytokines, reactive oxygen species, macrophage infiltration, adipocyte imbalance, or vascular damage; these effects are associated with metabolically active tissues such as adipose tissue, skeletal muscle, and the liver, implicating CLGI in metabolic diseases [1,31]. Age-related immunosenescence and the accumulation of cellular debris are also part of CLGI evolution in older people [32,33].

Estimating CLGI via different biomarkers could pave the way for its early detection. But aside from the key discoveries in recent decades describing the expression patterns of some CLGI biomarkers which have cemented its pathophysiological functions, the diagnosis of CLGI remains to be defined from a technical perspective. While biomarkers of CLGI have been identified, their use is hampered by, on the one hand, a lack of basal values, and on the other, detailed knowledge of their levels in a pathological context. A cluster or panel of biomarkers could best describe CLGI, and in the following section we present the biomarkers currently under investigation, with a brief description of those from human studies, and a focus on murine models.

## 3. CLGI Biomarkers

### 3.1. Human Studies

Susceptibility to, and the diagnosis, prognosis, and treatment of several diseases have all benefitted from fundamental and clinical research discoveries of biomarkers. In essence, a biomarker is a physiological, physical, molecular, or elemental indicator of a normal or pathogenic process or response. Biomarkers of CLGI so-far investigated are molecules at the local (e.g., tissue-specific) or systemic (e.g., blood) levels. However, as we shall see, the commonalities between the different inflammation statuses (i.e., AI, CI, CLGI) complicate the use of a single diagnostic biomarker specific to CLGI. 

Cytokines, adipokines (e.g., leptin), and chemokines are important signals of inflammation but the occurrence of these biomarkers per se cannot distinguish one type of inflammation from another. In humans, along with the numerous CLGI causes, which inevitably lead to modulation of biomarker expression, a considerable inter-individual variation exists according to age, body weight, eating habits, smoking, or alcohol consumption [7]. With so many factors producing variability, a consensus on the best biomarkers for the assessment of the physiological role and prediction power of CLGI is far from being reached. Based on the discussions held by the International Life Sciences Institute and the Europe Nutrition and Immunity Task Force during the first half of the 2010s, Calder et al. [7] suggested that a robust strategy would be to investigate biomarker expression patterns, or the composition of biomarker panels or clusters, from molecules described in human clinical studies. The benefit of this approach is the breadth of its applicability, encompassing nutritional, neurological, and geriatric research. A single biomarker cannot robustly discriminate CLGI from other forms of inflammation, and this approach has a precedent in the various “omics” fields of research. 

A recent systematic review of inflammation biomarkers reported that the major inflammation signals currently being explored in human intervention studies are CRP, cytokines (notably IL-6 and TNF-α), adhesion proteins such as VCAM-1 and ICAM-1, and the MCP-1 chemokine [34]. CRP, for example, is a major AI biomarker, produced in the liver, with a proven record of clinical use and well-established protocols for its analysis and detection. A key feature of CRP is its responsiveness to inflammation stimuli, making it a sensitive biomarker of inflammation [35]. Elevated CRP, for instance, has long been used as a risk indicator of cardiovascular disease (CVD) in humans, and a threshold indicating moderate to high risk to CVD (>1.5 mg/L) is well-defined [35]. However, there are still no widely accepted, conclusive, local, or systemic biomarkers specific to CLGI forthcoming from human studies, nor any definition of the basal expression of potential biomarkers (which deserves particular attention).

### 3.2. Murine Model Studies

When it comes to CLGI research in rodents, a similar range of biomarkers to that in human studies is observed. The ensemble of the biomarkers explored in the literature is presented in Table 1, all of them emanating from studies that followed the chronic induction of LGI using AGE-rich diets, obesogenic diets, or the effects of metabolic endotoxemia. These biomarkers were reported in a series of organs including the brain, the adipose tissue, or the kidneys, and non-invasive analysis of feces has been also used to address CLGI in rodents.

Despite the fact that we have not attempted to be exhaustive in this review, significant diversity can be observed among the molecular biomarkers of inflammation listed in Table 1. Cytokines and chemokines are by far the most studied molecules, and IL-6, TNF-α, and MCP-1 figure among the most studied biomarkers in dietary studies with animal models. Both IL-6 and TNF-α are widely used in CLGI studies and are expressed in several cell types, while TNF-α is the most cited CLGI biomarker (Table 1). Both TNF-α and IL-6 are well-described cytokines with well-established analytical tools available for their analysis in various sample types (e.g., validated ELISA tests). It is worth noting that the most studied CLGI biomarker in humans, the CRP protein, is not widely studied in murine models. This is due to the very different behavior of this molecule in rodents compared to humans, where it works as an acute-phase protein—the typical range of rodent expression of CRP, from 5 to 9 mg/L with peaks at only 17 mg/L after AI stimulation with LPS as explored by Huang et al. [61]. Thus, while the transposition of CRP as a CLGI biomarker from rodents to humans is limited, it highlights the need for CLGI biomarkers with similar responsiveness in different taxa.

Among the publications listed in Table 1, several adopted the relative analysis of the expression of several cytokines’ genes. Besides the usefulness and importance of such techniques, a more quantitative approach would better address the basal and pathologic levels of certain biomarkers, facilitating the construction of diagnostic biomarker clusters or panels. For example, independent studies on the circulating levels of the anti-inflammatory cytokine IL-10 have described similar basal levels of this cytokine, ranging from 10 to 15 pg/mL [37,39].

A further issue in the research of some CLGI biomarkers is the differences in the expression of certain molecules across publications. For example, the CLGI-induced disruption of adhesion molecules such as ICAM-1 and VCAM-1 is reported to result in their overexpression by different publications investigating high-fat diets (HFD) or AGE consumption [47,48,49]. Both biomarkers would therefore seem to have a concordant and repeatable expression pattern as a result of inflammation. On the other hand, expression of the RAGE receptor or the transcriptional factor NF-κβ has been reported to be both up- and down-regulated as a result of CLGI, depending on the tissue being studied (Table 1). The following sections summarize current efforts, notably from the last two decades, on the characterization of CLGI, its putative biomarkers, and its proposed physiological roles in murine models.

## 4. High-AGE Diets and CLGI Initiation in Murine Models

Advanced Glycation End-Products (AGEs) are the result of non-enzymatic, post-translational reactions between reducing carbonyls and protein-amino groups, nucleic acids, or aminophospholipids [62]. The formation of AGEs by the Maillard reaction gained prominence in human health research during the 1950s, after the discovery of glycated hemoglobin under physiological conditions and its reported correlation with glycemic levels in diabetic patients [63]. The glycation of cellular proteins has a negative effect on cell and tissue function, molecular aging, and chronic disease development [64,65]. In addition to their endogenous occurrence, AGEs are also formed during the thermal processing of foods, significantly increasing humans’ exposure to dietary AGEs (dAGEs). Food-borne AGEs are part of a heterogeneous group of chemically stable molecules resulting from the Maillard reaction, some of which are implicated in benignly improving flavors, aromas, and browning, while others are thought to be involved in adverse health effects (e.g., chronic inflammation, degenerative diseases, aging, insulin resistance) [64,65].

AGEs have a close relationship with inflammation and oxidative stress which is mediated by RAGE, their eponymous receptor which is part of the immunoglobulin superfamily and participates in immune surveillance in the lungs, liver, vascular endothelium, monocytes, dendritic cells, and neurons, to name only the major locations identified so far [66]. RAGE is a promiscuous receptor for which multiple ligands have been identified (e.g., HMGB1, S100, multiple AGEs), making it an important pattern recognition receptor (PRR) and inflammation trigger [3]. Activation of the RAGE-AGE axis has been described as a key mechanism leading to the production of pro-inflammatory cytokines, which leads to the maladaptive tissue remodeling caused by the modulation of genes and proteins implicated in extracellular matrix composition, cellular connectivity, elasticity, and tissue flexibility [67]. In this way, the long-term consumption of dAGEs would expose the immune system to CLGI activation [68].

dAGEs have been proposed as key factors in triggering inflammation in both healthy and diabetic individuals [69]. Among the AGEs already identified and characterized, carboxymethyl-lysine (CML), a glycation product of lysine residues, is the most studied [70,71]. Dietary sources of CML are diverse and include processed meat, dairy products, infant formulas, instant coffee, and biscuits [72]. For instance, the average content of dietary CML (dCML) in infant formulas is 70 times greater compared with maternal milk. For adults, based on the European diet rich in bread and coffee, the minimal daily consumption of dCML is estimated at 5 mg/day (per body weight) [73]. A large body of evidence points to the implication of CML and other AGEs in the inflammatory aggravation of obesity, diabetes, delayed wound healing, or ovarian hormone dysregulation in rodent models. Table 2 presents several papers on the investigation of the inflammatory effects of different AGE-enriched diets on health in murine models.

Protein-bound CML is a high-affinity RAGE ligand [83,84]. The pro-inflammatory effect of dCML has been demonstrated in a comparison between wild-type and RAGE knockout animals receiving a CML-enriched diet (50, 100, and 200 µg CML/g_food_). In wild-type animals receiving dCML, a dose-dependent increase in expression of VCAM-1 was observed, both histologically and in mRNA expression, while RAGE expression was increased significantly only at the protein level. The RAGE knockout animals were apparently protected from an increase in these inflammation triggers, and no significant VCAM-1 expression increase with dCML dose was reported in this genotype [49]. Such a protective effect over RAGE knockout animals was previously demonstrated in obese male mice that received both fat and AGE enriched diet. Harcourt et al. [80] reported that MCP-1 levels, both in plasma and kidneys of RAGE knockout mice, were reduced followed by improved MIP (macrophage migration inhibitory factor) level in the same samples. Further, the influence of the RAGE-AGE axis upon the promotion of inflammation was highlighted by the application of alagebrium, an AGE cross-link breaker currently investigated as an anti-AGE compound [85]. In animals receiving alagebrium, MCP-1 and MIP levels behaved in the same way as in RAGE knockout in addition to improved glycemic control [80]. The potential deleterious effect of dCML can also be inferred from the endocrine perspective, as reported in experiments on mice with ovarian hormone dysfunction. Thornton et al. [55] compared the effect of a CML-enriched and low-AGE (L-AGE) diet on ovary dysfunctions in C57BL/6 mice. The results, after 13 weeks, from mice receiving the high-AGE (H-AGE) diet showed a dysregulation of the estrous cycle and superovulation followed by an upregulation of macrophage marker F4/80 mRNA expression. The local expression of macrophage biomarkers was significantly lower in animals in receipt of a low-AGE (L-AGE) diet. Other CLGI biomarkers were examined in the ovarian tissues, with the expression of pro-inflammatory cluster of differentiation 11 (CD11) increasing and anti-inflammatory CD206 decreasing among the H-AGE mice. This may be explained by the different RAGE expression among the follicular cell types in response to gonadotrophins, since dAGEs can interfere in the gonadal cycle [86]. Chatzigeorgiou et al. [51] reported that peripheral blood mononuclear cells (PBMCs) isolated from female mice receiving an H-AGE diet had both RAGE and scavenger receptor type A (SR-A) downregulation which could be involved in dAGEs accumulation in endocrine tissues as the ovaries.

Similar to observations in animals fed HFD, dAGEs may also play a role in remodeling of gut microbiota. Evidence from experiments on rats and C57BL/6 mice demonstrated that dietary MG-H1 had a pro-inflammatory effect as well as remodeling gut microbiota [39,75]. Diet-induced gut permeability was demonstrated to result from increased CML, CEL, and MG-H1 consumption on Sprague-Dawley male rats exposed to a baked chow diet for 24 weeks. The prolonged exposure to AGEs led to kidney injuries associated with chronic kidney disease (CKD), increased MCP-1 levels in plasma, and the activation of the complement system as measured via C3a and C5a biomarkers. Such inflammatory status was associated with the dysregulation of intestinal permeability. In the high-AGE-diet group, occludin and claudin-1 gene expression were downregulated and increased circulating levels of LPS were evidenced compared to healthy animals. However, the deleterious effect of the overconsumption of AGEs was reversed with alagebrium administration or high fiber ingestion, being this last one associated with C5a proinflammatory effector decrease in *db*/*db* mice (more insights on the dietary modulation of gut microbiota and permeability are discussed ahead) [75]. MG-H1 is a methylglyoxal glycation product of arginine residues [39]. Regular animal food pellets supplemented with 15 μmol MG-H1/g_food_ produced a significant change in glucose metabolism, followed by increased expression of pro-inflammatory cytokines IL-1β, IL-17, TNF-α, and a decrease in anti-inflammatory cytokines IL-10 and IL-6 over 22 weeks treatment. The influence of dietary MG-H1 on gut microbiota was evidenced in reduced butyrate-producing species such as *Candidatus* Arthromitus and *Anaerostipes* sp. in animals receiving higher levels of MG-H1 [39]. From the experimental point of view, studies such as these have the benefit of using more accurate technologies (e.g., HPLC-MS/MS) for the identification and measurement of protein modification in laboratory-made diets [39,49]. Previously, the AGE enrichment of diets by autoclave, for instance, would result in the formation of several glycation products, making it difficult to attribute the effects of a single target molecule. 

An analytical approach employing CML isotopes has demonstrated that dCML accumulates primarily in the kidneys, but also in the ileum, colon, and lungs in a RAGE-independent manner [87]. RAGE is important in triggering respiratory allergies, being involved in complications of lung cancer, asthma, and bronchoalveolar inflammation [88]. To investigate the involvement of the AGE-RAGE axis in respiratory inflammation, mice were exposed to an H-AGE diet over 4 weeks and the bronchoalveolar lavage was analyzed. This presented higher polymorphonuclear (PMN) cells, cytokines (IL1B, IL-6, and MM1, MMP-2), and TNFsRII, all of which may contribute to aggravation of lung injury, and which were associated with the triggering of inflammation by circulating dAGEs. Here, then, the responsiveness of the lungs to dAGEs is likely to be associated with pulmonary RAGE expression [89].

A striking characteristic of dAGEs is their interference in the physiology of multiple organs and tissues. In addition to their influence on the lungs, wound healing has also been shown to be disrupted by dAGEs, especially when associated with diabetes [90]. Diabetic mice with a skin injury receiving an H-AGE diet had slower vascularization, epithelialization, and local inflammatory cell infiltration than controls receiving an L-AGE diet [82]. These wound healing rates were further correlated with increased plasma HMGB1 expression in another study on Kunming mice, a model of age-related decline [60].

However, a question that remains to be answered concerns the reversibility of the several physiological effects of AGE consumption as previously presented in this topic. A recent study published by Dongen et al. (2021) [74] demonstrated that the effects of a high-AGE diet (baked chow diet) were able to shift the gut microbiota composition and promote a pro-inflammatory status in C57BL/6 female mice. These animals presented a significant increase in the circulating levels of free CML, MG-H1, and carboxyethyl-lysine (CEL) after a 10-week exposure to a modified diet compared to control animals in a regular diet. However, both components (including the inflammatory biomarkers and gut microbiota structure) were reversed when a non-baked diet was introduced in week 5, replacing the high-AGE diet. Withal, some light still remains to be shed over longer-exposure protocols where a chronic induction of the inflammatory factors could be distinguished.

## 5. Other Diet-Induced CLGI in Murine Models

### 5.1. Obesogenic Diet-Induced CLGI 

The underlying role of inflammation in obesity has been discussed for over 20 years [20]. Table 3 summarizes the recent evolution of CLGI studies of high-calorie diets, including HFDs and high-carbohydrate diets (HCDs) To date, as a multifactorial disease, obesity is recognized as a metabolic dysfunction with a strong CLGI factor related to an increase in adipose tissue and hypothalamic dysfunction [91,92]. The relationship between excessive macronutrient intake and inflammation relies upon the promotion of immune cell infiltration in the adipose tissue, in the hypothalamus (the core site on appetite control), as well as the liver [93,94]. Macrophages are central to the development of obesity-induced CLGI, in addition to several other mechanisms which promote inflammation in this context (e.g., hypoxia, inflammasome activation) [13]. White adipose tissue (WAT) is no longer considered to be an inert tissue, and has been shown to participate in metabolic dysfunction linked to inconspicuous inflammation with a low-grade release of inflammatory mediators, as witnessed by the increased expression of inflammation biomarkers in obese animals (e.g., TNF-α, MCP-1, IL-6) [19]. In corroboration, the promotion of the pro-inflammatory state in an obese individual is related to their increase in adipose tissue, made manifest when adipokine (e.g., adiponectin, leptin) levels were demonstrated to vary with weight gain [95].

Obesogenic diets have been shown to increase CVD risk in humans as well as comorbidities classically associated with being overweight [97]. A closer look at diet-induced obesity (DIO) studies in murine models suggests its involvement in the aggravation of CLGI. DIO promotes alterations in metabolic tissues such as macrophage infiltration, adipocyte hypertrophy, and death, as well as the activation of proinflammatory pathways within the WAT and the hypothalamus. Macrophages and lymphocytes are part of the heterogeneous composition of adipose tissues [98]. Adipocyte hypertrophy is followed by early-stage adipocyte cell death, accelerating the abnormal recruitment of bone-marrow macrophages and amplification of immune cell responses [99]. In light of important histopathologic evidence derived from obese mice, a greater density of macrophages was shown to surround dead adipocytes [100]. A 4-fold incidence of macrophages was demonstrated to occur in the adipose tissue followed by an increase in MCP-1 (a macrophage chemoattractant) in obese mice compared with lean animals [101,102]. Special attention was paid to the in situ expression of cytokines and demonstrated that macrophages and adipocytes participate in the augmented expression of adipokines, especially IL-6, leptin and adiponectin, in addition to a reduced blood supply and increased hypoxia and oxidative stress in WAT, all of which contribute to systemic inflammation and insulin resistance [103].

It appears that under a chronic consumption of an HFD, adipose tissue promotes an increase in systemic cytokine production. Heijden et al. [37] observed that after 24 weeks, mice receiving an HFD showed noticeable adipose tissue inflammation, but hepatic inflammation signals were only detectable after 40 weeks. The temporal expression of TNF-α, MCP-1, and macrophage F4/80+ receptors described in mice suggests that adipose tissue inflammation precedes hepatic inflammatory response to HFDs. At least one other study demonstrated the effect of an HFD on CLGI in the liver. A recent publication by Guerra and colleagues [38] demonstrated that, as described for adipose tissue and the brain, the consumption of an HFD (60% fat) by C57L/6 female mice for 24 weeks promoted the infiltration of macrophages and mononuclear cells into hepatic tissue. The latter finding demonstrates that the hepatocyte expression of anti-inflammatory and pro-inflammatory biomarkers, respectively, IL-10 and TNF-α genes, increased significantly [38]. On the other hand, IL-1β and IL-6 (pro-inflammatory) remained at the same levels compared with the control diet group, demonstrating a heterogeneous effect of this type of diet on different cytokines [38]. 

A significant limitation for the understanding of the real effects of diet-induced CLGI is confounding factors such as the variable quality of fat among the different commercially available diets for animal experiments. It appears that not only the duration of the exposure to an HFD is important, but also its composition. Benoit et al. [41] hypothesized that the difference in fat quality among diets could influence the effects observed in the scientific literature. They tested diets including a regular chow, low-fat diet (cLFD; 8% fat), a semisynthetic low-fat diet (sLFD; 12% fat), and a semisynthetic HFD (sHFD; 40% fat). Differences observed among experimental groups were related to the origin of the fat component, but not the amount of fat per se. Interestingly, for several of the physiological features evaluated, the two LFDs yielded apparently contradictory results. The insulin levels in plasma, for instance, were significantly higher in the sLFD animals, while no difference was evident between regular cLFD or sHFD groups. Similarly, MCP-1 expression in the epididymis WAT significantly increased only in the 40% fat, sHFD treatment. Pro-inflammatory IL-6 expression was increased only in animals receiving the sLFD, highlighting how heterogeneous the dietary stimulation of a low-grade inflammation response may be with diets of different quality and composition [41].

Reporting similar findings on the effects of macrophages in adipose tissue, some recent studies have demonstrated the role of macrophage infiltration in the hypothalamus. As stated above, the hypothalamus is a fundamental actor in appetite control, and its proper function is therefore required to maintain energy homeostasis [104]. Remarkably, excessive macronutrient intake induces the infiltration of macrophages to the hypothalamic core, which is involved in sensitivity to peripheral levels of glucose and metabolism signals such as leptin, insulin, and free fatty acids (FFA) [105]. Lainez et al. [5] demonstrated that an HFD doubled macrophage infiltration to the hypothalamus of obese male mice compared with controls in a regular diet. A second study examined the production of cytokines in the hypothalamic arcuate nucleus, and the overexpression of pro-inflammatory cytokines was demonstrated to be related to a loss of glucose homeostasis coordination in a male mouse model of inflammation (LysM) [24]. In contrast, a transcriptomic approach was used to study the abnormal immune responses of the hypothalamus and demonstrated that inflammation was not associated with HFD consumption. Based on a 4–8-weeks animal experiment including wild type, TLR4 knockout, and CD14 knockout animals, no significant difference in inflammation transcripts was observed among animals of different genotypes receiving an HFD [4]. Both the TLR4 and CD14 receptors are part of the innate immune system, participating in PRRs recognition. This is important because TLR4 and CD14 cooperate in the pro-inflammatory response to exogenous stress factors such as LPS, as described below [106].

### 5.2. Diet-Induced Gut Microbiota Remodeling and CLGI: A Mechanism of Metabolic Endotoxemia

The gut microbiota is an important functional unit established in the gastrointestinal system (GIS) that has co-evolved to participate in the regulation of host homeostasis, in addition to its direct role in digestion [107]. The colonization of the GIS by microbiota begins at birth, but its composition is constantly remodeled throughout life according to the host’s genetic profile and environmental conditions (e.g., diet, smoking) [108]. The gut microbiome comprises a vast community of trillions of microorganisms, the most numerous of which are ensembles of both Gram-negative and Gram-positive bacteria, and which include a relatively minor component of fungal species [109]. The structure of gut bacterial communities comprises two main phyla: Bacteroidetes (mostly Gram-negative) and Firmicutes (mostly Gram-positive), and the proportion of last increases with age [110,111]. Humans and mice have a broadly similar Firmicutes/Bacteroidetes (F/B) ratio, with a predominance of Bacteroidetes over Firmicutes in healthy individuals in adulthood [112]. The prerequisite for a healthy microbiota is an adequately diverse and sufficiently dense microbial community. The intestinal bacterial community underlies important events in both the initiation and progression of CLGI as a result of unbalanced diet [33].

Diet is a major environmental factor in the remodeling of the gut microbiome [113] (Table 4). This much is apparent in comparative metagenomic studies, where a lower overall microbial diversity is observed in mice undergoing DIO protocols [114]. Several papers have described a shift in the F/B ratio in mice under HFD, with prominent Bacteroidetes cell death leading to a predominance of Firmicutes taxa [115,116]. Dysbiosis is linked to CLGI owing to the resultant increased permeability of the mucosal barrier in the gut to endotoxins (e.g., LPS), promoting a state of metabolic endotoxemia [115]. Diet-induced gut permeability results from the activation of mast cells and tight junction disruption, both associated with the production of tryptase and cytokines’ cascade activation [117]. Once gut epithelial permeability is increased, LPS may enter the bloodstream via both diffusion and chylomicrons, inducing a state of metabolic dysfunction mediated by TLR-4, RAGE, and CD14 receptor activation [118]. The causative link between circulating LPS and obesity has been illustrated by the remarkable CLGI in the hypothalamic region and concurrent, intensive local macrophage infiltration [24]. It is worth remembering that LPS is a gram-negative, cell wall glycolipid. Therefore, beyond the decrease in LPS producing groups in obese mice, such observations raise the possibility that the F/B ratio may not directly explain endotoxemia in obesity and CLGI. Magne et al. [119] proposed that, besides the overall reduction of Bacteroidetes groups in obese subjects, other Gram-negative taxa such as the Proteobacteria and Verrucomicrobia phylum remain important producers of LPS.

A considerable body of work has addressed the effects of HFD in reshaping the gut microbiome and in metabolic endotoxemia. Cani and colleagues [115] were the first to evidence LPS of gut microbial origin as a trigger of metabolic impairment. Accordingly, obese mice and lean controls presented different gut microbiota compositions and associated inflammation and metabolic dysregulation. The consumption over 4 weeks of an HFD by C57BL/6 male mice (12-weeks-old) doubled the circulating levels of LPS, and a concomitant increase in TNF-α, IL-1, IL-6, and PAI-1 levels was observed in the liver, visceral and subcutaneous fat, and muscles. A 4-week follow-up study on 12-week-old C57BL6/J mice fed an HFD also described a 2-fold increase in circulating levels of LPS (from 3 to 6 U/mL) compared with animals fed a regular, nutritionally balanced diet [121]. Some years later, a comparative analysis between germ-free mice and conventional pathogen-free mice pointed to clear evidence of the gut microbiota’s contribution to obesity and inflammation triggers. In this scenario, no metabolic dysfunction was observed in germ-free mice fed an HFD for 16 weeks, while TNF-α expression increased (concomitantly with the development of obese and diabetic phenotypes) in the conventional animals receiving the same HFD [122]. Later, a more comprehensive study of the effects of different diets on the gut microbiota was published. Serino et al. [57] compared the consumption of a classical HFD and an HFD enriched with fiber (galactooligosaccharides—HFD-GOS) during 12 weeks in C57BL/6 male mice. Animals receiving the conventional HFD presented an inversion of the F/B ratio, while those in receipt of the fiber-supplemented HFD-GOS retained an abundance of 90% for Bacteroidetes. The lower F/B ratio signature in the former was associated with higher circulating levels of LPS than the HFD-GOS group. In agreement with the increased circulating levels of LPS, plasma levels of IL-6, NF-kB and PAI-1 were also significantly higher in the HFD animals compared with the HFD-GOS group. Finally, the diabetic metabolic profile which developed in the HFD group was accompanied by increased paracellular permeability of the ileum, caecum, and colon, while a protective effect was observed with GOS supplementation [57].

Other consequences for gut homeostasis have been observed in DIO experiments in mice. Food fermentation by the gut flora plays a fundamental role in the production of metabolites such as short-chain fatty acids (SCFAs). Propionate, butyrate, and acetate are important SCFAs that play anti-inflammatory and immunoregulatory roles in the gut [123]. Butyrate is an important SCFA, originating from the fermentation of fibers, and a major source of energy for the microbial community in the gut. At the host level, butyrate acts as the epigenetic activator of gluconeogenesis, thus playing a role in energy metabolism in several cell types (e.g., hepatocytes, adipocytes), but mainly by influencing enterocyte energy homeostasis and epithelial integrity [123,124]. One effect of an HFD in mice is the diminution of butyrate-producing species (i.e., Ruminococcaceae and Lachnospiraceacae families) [125]. The negative effects of increased intestinal permeability are enhanced by the HFD’s effect on pro-inflammatory cascade triggers which actively promote cytokine expression (TNFα, interleukin IL-1β, IL-6, interferon γ (IFNγ), further disrupt tight junctions, and modify mucus composition at the surface of the intestinal epithelium. These factors combine to promote mucosal hyper-sensitization, inflammation, and concomitant villous atrophy [126]. IL-1β, for instance, was demonstrated to be a key molecule in the in vivo permeability control of gut epithelial cells. In C57BL/6 mice, an increase in IL-1β has been associated with NF-κB activation provoking an inflammatory cascade that in turn resulted in the overexpression of enterocyte myosin light-chain kinase (MLCK), at both the mRNA and protein levels [127]. In addition, an unbalanced diet may promote cytoskeletal cell modifications and epithelial cell apoptosis. Microtubule-actin cross-linking factor-7 (ACF7), for instance, participates in cytoskeleton dynamics and plays an important role in epithelial recovery. A study of HFD-fed ACF7 knockout mice revealed a disrupted intestinal homeostasis and a higher apoptotic level in the gut compared with wild-type mice fed the same diet [128]. Modifications of MLCK and ACF7, together with decreases in occludin, ZO-1, and claudin protein content in tight-junctions, support the theory that cell structure damage is related to epithelial impairment resulting from consumption of an HFD [129].

Considering the relevance of LPS to CI triggers, a cornerstone of the investigation of CLGI is the use of subclinical doses of LPS to mimic metabolic endotoxemia in a murine model [130,131]. In this way, LPS is used as a model molecule for the experimental initiation of systemic CLGI. The effects of administering subclinical doses of endotoxin in murine models have been examined in studies of the impacts of chronic and low-grade inflammation in obese phenotypes, including neuroinflammation and appetite control. The hypothalamus takes part in regulating food intake and body weight. In an investigation of the induction of hypothalamic CLGI, male Wistar rats were periodically injected with subclinical doses of LPS (100 µg/kg) via intraperitoneal injection. Animals chronically exposed to LPS (six doses over 24 h) became resistant to the hypophagic effect (reduction in feeding behavior) commonly observed as a result of exposure to LPS. These animals presented a 2-fold increase in hypothalamic phosphorylated c-Jun N-terminal kinase (JNK) levels compared with controls on saline injections [58]. JNK is a cell stress signaling marker regulating cell survival, but in the context of obesity and hypothalamic dysfunction it is involved in insulin receptor substrates (IRS) and recruitment of protein kinase B (AKT) and insulin-dependent translocation of forkhead box protein O1 (FoxO1) [132]. Chronic exposure to LPS was also shown to affect the phosphorylation of AKT, block IRS1 and impair the translocation of FoxO1 in hypothalamic GT1-7 mouse cells [58]. In Sprague-Dawley male rats, LPS administration was found to affect the brain via microglia hypertrophy, and ionized calcium-binding adapter 1 (Iba1) was reported to be overexpressed in immunostaining assay of brain tissue [59].

In addition to these findings, subclinical doses of LPS have been shown to affect the liver and adipose tissue. In male ApoE knockout C57BL/6J mice, the simultaneous administration of super-low doses of LPS with administration of an HFD resulted in the development of nonalcoholic fatty liver disease (NAFLD) steatohepatitis, followed by intense leukocyte infiltration and sustained expression of p38 mitogen-activated protein kinase (MAPK) [52], a molecule involved in the production of inflammatory mediators such as TNF-α and cyclo-oxygenase 2 (COX-2). Hepatocyte apoptosis was also associated with local cellular oxidative stress. Myeloperoxidase, an indicator of ROS production, was three times greater after 4 weeks of LPS exposure and was accompanied by an increase in hepatic expression of pro-inflammatory biomarkers IL-6, MCP-1, and TNF-α [52]. 

Research aimed at elucidating inflammatory patterns in the murine gut can therefore rely upon measures of the cytokines TNF-α, IL-6, IL-1, NF-kB, and IL-1β levels in animals receiving fat-rich diets, together with a temporal increase in systemic LPS and modified F/B ratios. Investigations which analyze these patterns may prove to be powerful predictive tools of CLGI. More recently, another useful metabolic inflammation biomarker has received attention in studies of intestinal microbial dysfunction mediation. Lipocalin-2 (LCN2) is a glycoprotein secreted by several cell types but was initially identified in activated neutrophils [133]. LCN2 is readily detected in serum, feces, and adipose tissue in the context of inflammation dysfunction. In the gut, the main sources of LCN2 are myeloid and intestinal epithelial cells—the same cells which are affected by the mucosal barrier dysfunction and increased permeability of the gut epithelium previously described. The responsiveness of LCN2 in the gastrointestinal tract was first demonstrated in colitis-induced models. Colitis has a close relationship with dysfunctional microbiota and permeability of the intestinal epithelium. Murine models of colitis have been adapted to relatively mild protocols in order to study the effect of low-grade endotoxemia on LCN2 expression. Chassaing et al. [56] used 8 days of subclinical doses of dextran sulfate sodium (DSS), an epithelial disruption promoting agent, to study the induction of intestinal CLGI in C57BL/6 mice. Low doses of DSS (0.25 and 0.5%) produced an increase in IL-1β mRNA, as well as significant ulceration of colonic epithelium observed by histopathology. Interestingly, while a dose of 0.25% DSS induced an increase in LCN2 levels in colon samples as well as in feces, no difference was observed in serum samples, but significant from 0.5% DSS injections. From the clinical point of view, the authors suggested that fecal LCN2 could be a sensitive, non-invasive biomarker of CLGI, and one that also has a close relationship with intestinal dysfunction [56].

## 6. Perspectives and Conclusions

Based on the data presented in this review, the involvement of diet in the induction and progression of CLGI has been clearly demonstrated in murine models. Since multiple factors which contribute to CLGI initiation probably occur simultaneously (e.g., higher AGE concentration in the circulation, endotoxemia), a better understanding of the interaction of inflammation biomarkers in different organs and tissues would help to elucidate key biomarkers of CLGI, as well as identify the most susceptible among them, pinpointing the potential physiological implications. The murine models described in this review have shed significant light on CLGI, particularly the importance of local (tissue level) measurements of CLGI biomarkers. The use of murine models is also helping to illuminate CLGI crosstalk among different organs due to the ease of sample collection, preparation, and analysis compared with other animal models or clinical studies. Indeed, significant advances have been made in the attribution of the physiological effects of CLGI, and the identification of potential biomarkers induced by target compounds such as dAGEs and dietary lipids in these models. They have enabled the study of direct effects of key dietary influences on animal physiology, but some limitations on the translational use of future biomarkers in clinical applications between murine models and humans may arise (e.g., CRP). In the future, the current use of qualitative or semiquantitative techniques for CLGI biomarker analysis should give way to more extensive and specific analytical techniques, such as proteomics, transcriptomics and metabolomics, that could drive research on both screening and quantification of CLGI biomarkers in the whole organism.

From the nutritional perspective, strategies for the prevention of CLGI are required to reduce the impacts of several metabolic diseases, as well as to promote healthy aging. A balanced diet is often recommended to maintain good health [134], and evidence is also emerging from murine models that CLGI may be attenuated through the use of natural products, hinting at the possibility that dietary intervention may have the potential to limit initiation and/or progression of CLGI. Indeed, the use of probiotics or extracts of certain berries has been reported to ameliorate diet-induced CLGI in murine models under HFD [135,136,137], though these studies require confirmation in clinical studies in humans. 

Lastly, current research on diet-induced CLGI has highlighted the utility of simultaneously examining several different inflammation biomarkers. Ongoing research has confirmed the involvement of several cytokines and other inflammation biomarkers in both the initiation and progression of CLGI, but their use for the prediction, and/or determination of CLGI still lacks consensus. We remain some way from defining a clinical diagnostic test for CLGI, but future CLGI treatment may target important receptors such as TLR4 and RAGE for the prevention of CLGI initiation, and diagnostics may rely upon the recent evolution of non-invasive biomarkers (e.g., lipocalin-2). With respect to the prospection of CLGI biomarkers, candidates should be systematically investigated to fill gaps in the whole-organism picture of inflammatory responses in different organs and tissues. From the diagnostics perspective, biomarker research should integrate current knowledge on the use of multiple biomarkers able to predict CLGI more robustly.

The overconsumption of AGEs (especially dCML) and obesogenic foods leads to several physiological effects which contribute to the onset of CLGI, and their study has contributed greatly to our understanding. Several different classes of biomarkers have been reported as important, but the levels of cytokines such as TNF-α and IL-6, as well as chemokines such as MCP-1 and adhesion molecules (e.g., VCAM1), are repeatedly cited as potential biomarkers in CLGI research in murine models. However, progress in this field can be only made with more robust analytical protocols. Particular attention should be paid to the quality and homogeneity of the dietary regimes employed, the simultaneous analysis of multiple organs under the same dietary protocol, and establishing both normal and pathological levels of key biomarkers already identified. Metabolomics studies that include multiple CLGI biomarkers have potential in this regard and offer the possibility of defining clusters or panels of biomarkers capable of specifically targeting this subtle, multi-layered and complex inflammatory process.

## Figures and Tables

**Table 1:** Diet-induced CLGI biomarkers across publications on murine models.

Role in CLGI	Class	Biomarker	Sample	Biomarker Levels between Control and Experimental Conditions (Arrows Show Expression Tendency)	References
Anti-inflammatory	Cytokine	Interleukin 2 (IL-2)	Brain	Control: 10 pg/mg Affected: 120 pg/mg (↑) (Protein)	[36]
			Liver	Control: 100 pg/mg Affected: 150 pg/mg (↑) (Protein)	
		Interleukin 10 (IL-10)	Adipose tissue	No change (→) (mRNA)	[37]
			Liver	No change (→) (mRNA)	[38]
					[37]
			Plasma	Control:10 pg/mL Affected: 20 pg/mL (↑) (Protein)	[37]
				Control: 15 pg/mL Affected: 9 pg/mL (↓) (Protein)	[39]
Pro and anti-inflammatory	Adipokine	Adiponectin	Plasma	Control: 90 μg/mL Affected: 60 μg/mL (↓) (Protein)	[40]
				Control: 30 µg/mL Affected: 45 µg/mL (↑) (Protein)	[41]
				Control: 8 ng/mL (→) (Protein)	[37]
	Cytokine	Interleukin 6 (IL-6)	Adipose Tissue	2-fold change (↑) (mRNA)	[41]
			BAL	No change (→) (Protein)	[42]
			Kidney	15-fold change (↑) (mRNA)	[43]
			Liver	No change (→) (mRNA)	[38]
				8-fold change (↑) (mRNA)	[44]
			Myocardium	Control: 21 ng/µg Affected: 28 ng/µg (↑) (Protein)	[45]
				18-fold change (↑) (mRNA)	
			Plasma	3-fold change (↑) (mRNA)	[46]
				Control: 4 pg/mL Affected: 2 pg/mL (↓) (Protein)	[39]
				No change (→) (mRNA)	[39]
Pro-inflammatory	Adhesion molecule	Intercellular adhesion molecule 1 (ICAM-1)	Aorta	1.4-fold change (↑) (mRNA)	[47]
			Myocardium	5.6-fold (↑) (mRNA)	[45]
			Plasma	3.5-fold change (↑) (Protein)	[48]
		Vascular cell adhesion molecule 1 (VCAM-1)	Aorta	2.5-fold change (↑) (Protein)	[49]
				1.4-fold change (↑) (mRNA)	
				2-fold change (↑) (mRNA)	[47]
			Kidney	4-fold change (↑) (mRNA)	[43]
	Cell receptor	Receptor for advanced glycation end-products (RAGE)	Aorta	2-fold change (↑) (Protein)	[49]
				No change (→) (mRNA)	
			Kidney	3-fold change (↑) (mRNA)	[50]
			Liver	3-fold change (↑) (mRNA)	[50]
				1.5-fold change (↑) (mRNA)	[44]
			PBMC	2-fold change (↓) (Protein)	[51]
			Spleen	3-fold change (↑) (mRNA)	[50]
	Chemokine	Keratinocyte chemoattractant (KC ou CXCL1)	BAL	Control: 10 pg/mL Affected: 600 pg/mL (↑) (Protein)	[42]
		Macrophage Inflammatory Protein 2 (MIP-2)	BAL	No change (→) (Protein)	[42]
			Liver	Control: 20 pg/mL Affected: 180 pg/mL (↑) (Protein)	[52]
			Plasma	Control: 900 pg/mL Affected: 1500 pg/mL (↑) (Protein)	[52]
	Cluster of differentiation	CD68 Clone ED1 (ED1)	Colon	4-fold (↑) (Histochemistry)	[53]
			Kidney	2.4-fold (↑) (Histochemistry)	[54]
		Cluster of differentiation 11 (CD11)	Ovary	1.2-fold change (↑) (mRNA)	[55]
		Cluster of differentiation 11 c (CD11c)	Adipose Tissue	No change (→) (mRNA)	[41]
		Cluster of differentiation 14 (CD14)	Plasma	Control: 400 ng/mL Affected: 800 ng/mL (↓) (Protein)	[41]
		Cluster of differentiation 206 (CD206)	Ovary	2.3-fold change (↓) (mRNA)	[55]
		Cluster of differentiation 43 (CD43)	Liver	4-fold change (↑) (mRNA)	[44]
		Cluster of differentiation 68 (CD68)	Adipose Tissue	No change (→) (mRNA)	[41]
			Plasma	4-fold change (↑) (mRNA)	[46]
		Cluster of differentiation 95 (CD95l or FAS Ligand)	Liver	2.5-fold change (↑) (mRNA)	[52]
			Plasma	4-fold change (↑) (mRNA)	[46]
	Complement system	Complement component 5a (c5a)	Plasma	3.5-fold change (↑) (Protein)	[48]
	Cytokine	Interferon gamma (IFN-γ)	Plasma	No change (→) (Protein)	[39]
		Interleukin 1 alpha (IL-1α)	Plasma	3.5-fold change (↑) (Protein)	[48]
		Interleukin 1 beta (IL-1β)	Adipose tissue	No change (→) (mRNA)	[37]
			BAL	No change (→) (Protein)	[42]
			Colon	2-fold change (↑) (mRNA)	[56]
			Liver	No change (→) (mRNA)	[38]
				2-fold change (↑) (mRNA)	[37]
			Plasma	Control: 1 pg/mL Affected: 5 pg/mL (↑) (Protein)	[39]
				2-fold change (↑) (mRNA)	[46]
		Interleukin 16 (IL-16)	Plasma	3.5-fold change (↑) (Protein)	[48]
				No change (→) (Protein)	[37]
		Interleukin 17 (IL-17)	Plasma	Control: 11 pg/mL Affected: 18 pg/mL (↑) (Protein)	[39]
		Tumor necrosis factor alpha (TNF-α)	Adipose tissue	3-fold change (↑) (mRNA)	[37]
			BAL	No change (→) (Protein)	[42]
			Brain	Control: 0.2 ng/mg Affected: 0.7 ng/mg (↑) (Protein)	[36]
			Kidney	10-fold change (↑) (mRNA)	[43]
			Liver	3-fold change (↑) (mRNA)	[38]
				No change (→) (mRNA)	[37]
				Control: 0.7 ng/mg Affected: 1 ng/mg (↑) (Protein)	[36]
			Myocardium	2-fold change (↑) (mRNA)	[45]
			Plasma	Control: 4 pg/mL Affected: 10 pg/mL (↑) (Protein)	[39]
				No change (→) (Protein)	[57]
		Tumor necrosis factor soluble receptor II (TNF-sRII)	BAL	Control: 150 pg/ml Affected: 849.4 pg/ml (↑) (Protein)	[42]
	Histologic hallmark (Dyong adipocytes and macrophages)	Crown-like structures (CLS)	Adipose tissue	Control: 0.4 CLS/mm^2^ Affected: 1.8 CLS/mm^2^ (↑) (Histochemistry)	[37]
	Inflammatory cell chemoattractant	Monocyte chemoattractant protein-1 (MCP-1)	Adipose tissue	2-fold change (↑) (mRNA)	[41]
				4-fold change (↑) (mRNA)	[37]
			Aorta	1.8-fold change (↑) (mRNA)	[47]
			BAL	Control: 10 ng/mL Affected: 90 pg/mL (↑) (Protein)	[42]
			Liver	2-fold change (↑) (mRNA)	[44]
				No change (→) (mRNA)	[37]
			Myocardium	3-fold change (↑) (mRNA)	[45]
			Plasma	4-fold change (↑) (Protein)	[48]
	Intracellular cell-signal	Factor nuclear kappa B (NF-κβ)	Brain	Control: 15 pg/mL Affected: 50 pg/mL (↑) (Protein)	[36]
			Liver	Control: 10 pg/mL (→) (Protein)	[36]
		IκB kinase (pIKK)	Peritone	4-fold change (↑) (mRNA)	[46]
		Mechanistic target of rapamycin (mTOR)	Liver	Control: 3 ng/mg Affected: 6 ng/mg (↑) (Protein)	[52]
		Protein kinase B (AKT)	Adipose tissue	1.5-fold (↓) (mRNA)	[41]
				No change (→) (Protein)	[58]
	LPS presenting protein	Lipopolysaccharide binding protein (LBP)	Plasma	Control: 13 ng/mL Affected: 15 ng/mL (↑) (Protein)	[4]
	Macrophage biomarker	Chloroacetate esterase (CAE)	Liver	6-fold change (↑) (Histochemistry)	[38]
		Macrophage glycoprotein (MOMA-2)	Liver	Control: 57 Cells/Area Affected: 70 Cells/Area (↑) (Histochemistry)	[52]
	Macrophage receptor	EGF-like module-containing mucin-like hormone receptor-like 1 (EMR1 or F4/80)	Adipose tissue	4-fold change (↑) (mRNA)	[37]
			Liver	2-fold (↑) (Histochemistry)	[38]
				2-fold change (↑) (mRNA)	[37]
			Ovary	2.3-fold change (↑) (mRNA)	[55]
			Plasma	2.5-fold (↑) mRNA	[46]
	Macrophage scavenger receptor	Scavenger Receptor A (SR-a)	PBMC	2.5-fold change (↓) (Protein)	[51]
	Microglia marker	Ionized Calcium-Binding Adaptor Molecule 1 (IBA1)	Brain	1.25-fold change (↑) (Histochemistry)	[59]
	Mitogen-activated protein kinase	p-p38MAPK	Liver	2.67-fold change (↑) (Protein)	[52]
		c-Jun N-terminal kinase (pJNK)	Brain	2-fold change (↑) (mRNA)	[58]
			Liver	1.84-fold change (↑) (mRNA)	[52]
	Neutrophil gelatinase-associated	Lipocalin-2 (LCN-2)	Colon	95-fold change (↑) (mRNA)	[56]
			Feces	Control: 5 ng/mL Affected: 80 ng/mL (↑) (Protein)	[56]
			Plasma	Control: 100 ng/mL Affected: 300 ng/mL (↑) (Protein)	[56]
	NF-κβ p65 subunit	Transcription factor p65 (p65)	Myocardio	1.5-fold change (↑) (mRNA)	[45]
			Peritone	2-fold change (↑) (mRNA)	[42]
	Oxidative stress biomarker	Myeloperoxidase (MPO)	Colon	No change (→) (Protein)	[56]
	Plasminogen regulation	Plasminogen activator inhibitor-1 (PAI-1)	Plasma	Control: 15 pg/mL Affected: 2 pg/mL (↓) (Protein)	[39]
				Control: 2900 pg/mL Affected: 3100 pg/mL (↑) (Protein)	[57]
	RAGE ligand	High–mobility group box 1 (HMGB1)	Plasma	4 ng/mL (↑) (Protein)	[60]
		S100 A8/A10	Myocardio	Control: 0.3 ng/mg Affected: 0.7 ng/mg (↑) (Protein)	[45]
	Secretory serine protease	Serine protease inhibitor A3N (Serpina3n)	Brain	Control: 180 IOD Affected: 220 IOD (↑) (In situ hybridization)	[4]
	Signaling adapter	Insulin receptor substrate 1 (IRS-1)	Brain	No change (→) (Protein)	[58]
	Transport protein	Fatty-acid-binding Proteins (FABP)	Plasma	2.5-fold change (↑) (mRNA)	[46]

BAL: BronchoAlveolar Lavage; IOD: Integral Optical Density.

**Table 2:** Studies (in reverse chronological order) of CLGI induction by Advanced Glycation End-Products in murine models.

Diet	AGE Levels in the Diets (Technique)	Time of Exposure (Weeks)	CLGI Biomarkers	Target Organ/Animal Model/Sex	Reference
High-AGE diet	Control: CML: Free: 3.0 μg/g; protein-bound: 10.0 μg/g Carboxyethyllysine (CEL): Free: 0.4 μg/g; protein-bound: 2.1 μg/g MG-H1: Free: 0.4 μg/g; protein-bound: 89.0 μg/g Baked chow diet: CML: Free: 1.0 μg/g; protein-bound: 38.0 μg/g CEL: Free: 0.5 μg/g; protein-bound: 30.5 μg/g MG-H1: Free: 1.6 μg/g; protein-bound: 137 μg/g (UPLC-MS/MS)	10	CRP, TNF-α, IFN-δ, IL-6, IL-10	Plasma, fecal microbiota/C57BL/6/Females	[74]
High-AGE diet	Control: CML: 2.58 μg/g CEL: 0.89 μg/g MG-H1: 34.51 μg/g Baked chow diet: CML: 4.87 μg/g CEL: 1.38 μg/g MG-H1: 43.49 μg/g (QTRAP LC-MS/MS)	24	MCP1, LPS, C3a, C5a, occludin	Plasma and gut/Sprague-Dawley and C57BL/6/Males	[75]
MG-H1-enriched diet	3420 μg/g (HPLC-MS/MS)	22	IL-1β, IL-17, IFN-γ, TNF-α, PAI-1	Plasma, fecal microbiota/C57BL/6/Males	[39]
CML-enriched diet	Control: 61.9 μg/g CML diet: 605 μg/g (ELISA)	13	F4/80, CD11c, CD206	Ovary/C57BL/6/Females	[55]
CML-enriched diet	Commercial CML 0.1% w/w	24	C5a, ICAM, IFN-δ, IL-1α, IL-1β, IL-1ra, IL-6, IL-10, IL-12, IL-13, IL-16, IL-17, IL23, TNF-α	Plasma/Swiss/Males	[48]
High-AGE diet	Control: CML: 2.79 μg/g Baked chow diet: CML: 14.45 μg/g (HPLC)	6, 12, 18	Microbiota	Gut/Sprague-Dawley/Males	[76]
CML-enriched diet	Control: 17.5 µg/g CML diet: 200 μg/g (HPLC-LTQ)	36	VCAM-1, RAGE	Aorta/RAGE KO/Males	[49]
High-AGE diet	Control: Furosine: 28.80 μg/g Hydroxymethylfurfural (HMF): 0.44 μg/g CML: 2.20 μg/g Baked chow diet: Furosine: 1787.08 μg/g HMF: 5.15 μg/g CML: 12.46 μg/g (ND)	22	Microbiota	Gut/Wistar/Males	[77]
High-AGE diet	Control: Furosine: 28.8 μg/g HMF: 0.44 μg/g Bread crust diet: Furosine: 49.5 μg/g HMF: 4.26 μg/g (HPLC)	22	Microbiota	Gut/Wistar/Males	[78]
High-AGE methionine choline-deficient diet	Control: 31 nmol/g_lysine_ CML diet: 137 nmol/g_lysine_ (ELISA)	12	Il-6, MCP-1, RAGE, CD43	Liver/Sprague–Dawley/Males	[44]
High-AGE diet	Control: CML diet: 13 μg/g Fructoselysine: 104 μg/g Furosine: 268 μg/g H-AGE: CML diet: 760 μg/g Fructoselysine: 205 μg/g Furosine: 526 μg/g (ND)	12	RAGE, SR-A	PBMC/Wistar/Females	[51]
High-AGE diet	Control: 23 μg/g AGE diet: 110 μg/g (ELISA)	4	TNFα, TNF sRII, IL-1β, IL-6, IL-10, CXC, KC, MIP-2, CINC-1, MCP-1	Bronchoalveolar lavage/CD-1/Mixed	[42]
High-AGE diet	Control: CML: 60649 U/g H-AGE CML: 197305 U/g (ELISA)	39	Neutrophil infiltration	Liver/C57BL/6NHsd/Males	[79]
HFD-High-AGE diet	Control: 20.90 nmol CML/mol lysine/g AGE diet: 101.90 nmol CML/mol lysine/g (ND)	16	MCP-1, MIF (macrophage migration inhibitory factor), RAGE	Kidney/C57BL/6 (RAGEKO)/Males	[80]
Market bought High-AGE diet	53–1473 AU/g	1	HMGB1	Wound healing/Kunming mice/Males	[60]
High-AGE diet	Control: 1 µmol CML/lysine/day AGE diet: 4 µmol CML/lysine/day (ELISA)	16	IL-6, TNFα, ICAM-1, MCP-1, p65, RAGE, S1—A8/A9	Myocardio/RAGE KO/Males	[45]
High-AGE diet	Control: 112 µg/g CML diet: 785 µg/g (ELISA)	5, 9, and 13	Macrophage infiltration (ED1-positive), MCP-1	Kidney/Sprague-Dawley/Males	[54]
High-AGE diet	Control: 119,000 µg/g CML diet: 930,000 µg/g (ELISA)	11	Macrophage infiltration	Colon/Sprague-Dawley/Males	[53]
High-AGE diet	Control CML: 2700 U/mg CML diet: 12,500 U/mg Control MG: 0.65 U/mg MG diet: 2.5 U/mg (ELISA)	8	VCAM-1, RAGE, MOMA-2	Aorta/ApoE KO/Males	[81]
High-AGE diet	Control CML: 107 U/mg CML diet: 535 U/mg Control MG: 3.6 U/mg MG diet: 18 U/mg (ELISA)	28	Inflammatory cell infiltration	Skin/db/db/Females	[82]

ND: not described; MG: methylglyoxal.

**Table 3:** Studies of CLGI (in reverse chronological order) resulting from high-calorie intake interventions in murine models.

CLGI Trigger	Target Organ	Time of Exposure (Weeks)	CLGI Biomarkers	Animal/Sex	Reference
HFD	Liver	24	TNF-α, IL-1β, IL-6, IL-10, CAE^+^, F4/80^+^	C57BL/6J/Females	[38]
	Adipose tissue, liver	24, 40, and 52	TNF-α, IL-1β, MCP-1, F4/80^+^, crown-like structures	C57BL/6/Males	[37]
	Adipose tissue	11	CD14, AKT, CD68, C11c, MCP-1, IL-6	C57BL/6/Males	[41]
	Hypothalamus	8	Serpina3n	C57BL/6J, TLR4 KO, CD14 KO/Males	[4]
	Gut microbiota	12	NF-kB, mTOR, AKT	C57BL/6/Males	[57]
	Gut microbiota	8	PPARγ, C/EBPa, FAZ, aFABP, CD68, F4/80, p-IKK β, p65, TNF-α, IL 1β, IL-6	C57BL/6J, TLR4 KO C57BL/10ScNJ/Males	[46]
High-calorie diet (30% fructose)	Liver, brain	8	TNF-α, IL-2, NF-κB, HVA	Sprague-Dawley/Males	[36]
Intragastric fructose injection	Serum, liver, pancreas	20	IL-6, TNF-α, MIP-2, IL-10	Sprague-Dawley/Males	[96]

**Table 4:** Studies of murine gut microbiota and CLGI.

CLGI Trigger	Target Organ	Time of Exposure (Weeks)	CLGI Assessment Biomarkers	Strain/Sex	Reference
LPS	Hypothalamus	12	Iba1, TH	Sprague-Dawley/Males	[59]
	Hypothalamus	1	IRS1, AKT, JNK	Wistar/Males	[58]
	Liver	4	p38 MAPK, MPO, TNF-α, MCP-1, IL-6	ApoE KO, C57BL/6J/Males	[52]
	Plasma	8	TNF-α, TNF-β, MCP-1, IL-6	ApoE KO/Male	[120]
DSS	Colon, feces, plasma	1	Lipocalin-2	C57BL/6/Males; IL-10 KO/Females	[56]
